# The Heat Shock Response of *Mycobacterium Tuberculosis*: Linking Gene Expression, Immunology and Pathogenesis

**DOI:** 10.1002/cfg.183

**Published:** 2002-08

**Authors:** Graham R. Stewart, Lorenz Wernisch, Richard Stabler, Joseph A. Mangan, Jason Hinds, Ken G. Laing, Philip D. Butcher, Douglas B. Young

**Affiliations:** 1 Department of Infectious Diseases and Microbiology Faculty of Medicine Centre for Molecular Microbiology and Infection Imperial College of Science Technology and Medicine London SW7 2AZ UK; 2 School of Crystallography Birkbeck College University of London Malet Street London WC1E 7HX UK; 3 Department of Medical Microbiology St George's Hospital Medical School Cranmer Terrace London SW17 ORE UK

## Abstract

The regulation of heat shock protein (HSP) expression is critically important to pathogens such as *Mycobacterium tuberculosis* and dysregulation of the heat shock response results in increased immune recognition of the bacterium and reduced survival during chronic infection. In this study we use a whole genome spotted microarray to characterize the heat shock response of *M. tuberculosis*. We also begin a dissection of this important stress response by generating deletion mutants that lack specific transcriptional regulators and examining their transcriptional profiles under different stresses. Understanding the stimuli and mechanisms that govern heat shock in mycobacteria will allow us to relate observed *in vivo* expression patterns of HSPs to particular stresses and physiological conditions. The mechanisms controlling HSP expression also make attractive drug targets as part of a strategy designed to enhance immune recognition of the bacterium.


**Comparative and Functional Genomics**
*Comp Funct Genom* 2002; **3**: 348–351.
Published online in Wiley InterScience (www.interscience.wiley.com). **DOI:** 10.1002/cfg.183

**Conference Review**

### The heat shock response of *Mycobacterium tuberculosis*: linking gene expression, immunology and pathogenesis

Graham R. Stewart,[1]* Lorenz Wernisch,[2] Richard Stabler,[3] Joseph A. Mangan,[3] Jason Hinds,[3] Ken G. Laing,[3] Philip D. Butcher[3] and Douglas B. Young[1]

[1] *Department of Infectious Diseases and Microbiology, Faculty of Medicine, Centre for Molecular Microbiology and Infection, Imperial College of Science Technology and Medicine, London SW7 2AZ, UK*
[2] *School of Crystallography, Birkbeck College, University of London, Malet Street, London WC1E 7HX, UK*
[3] *Department of Medical Microbiology, St George's Hospital Medical School, Cranmer Terrace, London SW17 ORE, UK*

**Correspondence to: Graham R. Stewart, Department of Infectious Diseases and Microbiology, Centre for Molecular Microbiology and Infection, Imperial College of Science Technology and Medicine, London SW7 2AZ, UK. E-mail: g.stewart@ic.ac.uk*

Received: 31 May 2002
Accepted: 10 June 2002

### Abstract

**The regulation of heat shock protein (HSP) expression is critically important to pathogens such as *Mycobacterium tuberculosis* and dysregulation of the heat shock response results in increased immune recognition of the bacterium and reduced survival during chronic infection. In this study we use a whole genome spotted microarray to characterize the heat shock response of *M. tuberculosis*. We also begin a dissection of this important stress response by generating deletion mutants that lack specific transcriptional regulators and examining their transcriptional profiles under different stresses. Understanding the stimuli and mechanisms that govern heat shock in mycobacteria will allow us to relate observed *in vivo* expression patterns of HSPs to particular stresses and physiological conditions. The mechanisms controlling HSP expression also make attractive drug targets as part of a strategy designed to enhance immune recognition of the bacterium. Copyright © 2002 John Wiley & Sons, Ltd.**

**Keywords:** **microarray; transcriptional repressor; ANOVA; SAM; HspR; HrcA**

### Background

The classical heat shock proteins (HSPs) are stress-inducible molecular chaperones which represent the most conserved proteins in cellular life. In prokaryotes and eukaryotes their main role is to maintain a correctly folded and assembled protein component of the cell [14]. The essentiality of this function is reflected in the ubiquity of these proteins throughout cellular organisms. Indeed, it is the ancient nature of these highly conserved molecules, combined with the utility of their peptide-binding function, that has allowed evolution to engender many HSPs with functions additional to those of simple chaperones. Of greatest importance to the pathogen biologist are the roles of HSPs in the immune response. As host molecules whose expression is induced during stress conditions, such as infection with a pathogen, they are in perfect position to act as facilitators of the immune response, and nature has made no mistake in taking advantage of circumstance. Mammalian/host HSPs act as signals to the immune system through recognition by cell-surface receptors triggering inflammatory responses [2,8,9,33]. In addition, their functional role as chaperones has been utilized such that exogenous HSP carrying peptides from damaged or infected cells can, in an exquisitely efficient pathway, be taken up by receptor-mediated endocytosis into antigen presenting cells and processed for MHC class I-restricted presentation to T cells [1,3,4,10,29]. Thus, HSPs provide an important link between the innate and acquired arms of the immune response [27].

Copyright © 2002 John Wiley & Sons, Ltd.

What makes the expression of HSPs so interesting to the pathogen biologist is that pathogen HSPs are also recognized by specific receptors on host immune cells, triggering an inflammatory immune response [19,21,23,32]. In addition to this, the conservation between host and pathogen HSPs means that pathogen HSPs can be utilized by the host to shuttle peptides into the HSP-mediated antigen presentation pathway [17,30].

Thus, while the pathogen needs to increase expression of its HSPs in response to the stresses induced by host defenses [7,20,22,24], it must temper this need so as not to alert the host immune response to its presence. It is in the context of this dilemma that we are interested to learn how pathogens control the expression of their heat shock proteins. We have chosen to study *Mycobacterium tuberculosis*, an intracellular bacterial pathogen whose infection profile is critically governed by a dynamic relationship with the host immune response. In a recent study we demonstrated the vital importance of HSP regulation to *M. tuberculosis* by generating a mutant strain lacking the HspR repressor protein thus effecting dysregulation of the Hsp70 response [28]. The mutant strain constitutively overexpressed Hsp70 and associated HSPs, and during murine infection its survival was emphatically reduced. The underlying cause for this attenuation was enhanced immune recognition of the bacterium.

HSPs are so called because their expression is, in most cases, easily inducible by elevation of temperature causing denaturation of proteins. However, the expression of many is also determined by other stresses or environmental stimuli, such as oxidative stress, reactive nitrogen and micro-aerophilic conditions [13,18,26], to name several with particular importance to an intracellular pathogen. It is our aim to be able to describe under which conditions the mycobacterial HSPs are expressed and identify the molecular mechanisms behind their regulation.

### Strategy

We have embarked upon an approach based on whole genome expression profiling, using DNA microarrays to characterize the wild-type transcriptional response under relevant stresses/stimuli. These responses will then be dissected into their constitutive regulatory circuits by generating specific mutants in different regulatory pathways and comparing the expression profiles of resultant strains in stressed and unstressed conditions with the wild-type. Such an approach has only been made possible by the recent development of whole genome microarrays for *M. tuberculosis* and also by the acquisition of techniques to manipulate the genetics of the bacterium.

The microarray we are using consists of 3924 PCR amplicons (size range 60–1000 bp) derived from regions of all the predicted coding sequences of the sequenced strain of *M. tuberculosis* H37Rv [11], spotted onto poly-L-lysine-coated glass slides. RNA was extracted from bacteria grown in different conditions or from different strains, and cDNA was labelled with Cy3 or Cy5 dCTP during reverse transcription and competitively hybridized to the microarray. The analysis of microarray data is of critical importance and, like the technology itself, remains a largely developing science. We utilized two methods to analyse the data comparisons (e.g. wild-type vs. mutant strain or heat-shocked vs. non-heat-shocked cells). Firstly we used an ANOVA analysis, which took into account three main effects — the array effect, the gene effect and the variety (strain or environmental condition) effect, along with the pairwise interactions between these effects. The second method utilized was significance analysis of microarrays (SAM) [31], which is based on generating pseudo-datasets and assimilating sets of gene-specific $t$-tests in order to assign each gene a score on the basis of its change in expression relative to its own standard deviation. We are fortunate that heat shock is one of the best-studied prokaryote gene expression responses, as this endowed us with at least some biological knowledge to assess the performance of these analysis methods. In the wild-type heat shock vs. non-heat shock comparison, both analysis methods were able to identify all the known heat-inducible HSP genes as upregulated. There were other genes identified as upregulated and, although ANOVA and SAM largely concurred, there were a number of differences. Further experiments will be required to finally determine which analysis best reflects the true changes in gene expression.

### Heat shock proteins as part of a wider stress response

To date we have examined the transcriptional response of *M. tuberculosis* to heat shock at 45 °C

Copyright © 2002 John Wiley & Sons, Ltd. 

for 30 min. Of course, heat shock is a complex response which varies with both time and temperature, but this snapshot of the response provides a reference with which to compare transcriptomic changes in defined regulatory mutants. One general observation on the heat shock transcriptional profile is that the response is not simply the elevated transcription of the known HSPs but encompasses genome-wide changes in gene expression. Our first experiments to dissect this response involved gene knockout of two likely heat shock repressor proteins in *M. tuberculosis*, HspR and HrcA, which were identified by homology to regulators in *Streptomyces* and *Bacillus* [6,15]. We made mutants of both of these regulators using the pSMT100 gene replacement system [28]. Comparison of the expression profiles of strains lacking these regulators with wild-type, combined with identification of repressor binding sites in promoter regions, established the HspR and HrcA regulons. All members of these regulons were also found to be upregulated during heat shock and amongst them were many of the classical heat shock chaperones. Comparison of our results with those obtained in studies examining the regulation of other arms of the mycobacterial stress response reveals a high degree of crosstalk and overlap between the different stress regulons For example, the Hsp70 regulon forms a central element of the heat shock response and is under negative regulation by HspR in complex with Hsp70 itself [5,28]. However, the Hsp70 operon is also under control of the heat-inducible alternative sigma factor $\sigma$H [12], which in addition promotes transcription at the stress response sigma factors $\sigma$B and $\sigma$E [25]. Further to this, the functional activity of these sigma factors is under the control of anti-sigma factor pathways [16].

### Future work and conclusions

Thus, while we have identified how many of the major HSPs are regulated under heat shock, we have, in the process, revealed a greater complexity and extensiveness of the heat shock response than originally expected. Further disassembly of the response will require more extensive transcriptional profiling at different temperatures for different lengths of time and the generation of other regulatory mutants. It will also require the use of techniques to analyse post-transcriptional and post-translational control mechanisms of expression. It also remains to investigate the different types of stresses, such as reactive oxygen and nitrogen, which may stimulate HSP expression. Further identification of when during infection and in which tissues these proteins are expressed should enlighten us about what physiological stresses the bacteria are exposed to in these situations. HSPs make excellent candidates for such a study because they are extremely responsive to changes in stimuli, producing transcriptional changes of great amplitude. In addition, their qualities of high immunogenicity allow the possibility of using host immune recognition of different HSPs as a surrogate marker of HSP expression and in turn the physiological stresses imposed on the bacteria. Such knowledge could inform whether an infection was predominantly under aerobic or anaerobic conditions and would help greatly in the choice of treatment. Most importantly, we envisage that understanding the regulatory mechanisms behind mycobacterial HSP expression may allow the development of novel strategies for the treatment of tuberculosis. We have already demonstrated that dysregulation of the *M. tuberculosis* Hsp70 response allows the host to mount a more effective immunological response against the bacterium [28]. Thus, drugs that disrupt HSP regulation by interfering with specific regulators make an attractive mechanism by which to enhance host immunity.

### Acknowledgements

We thank Brian Robertson for help with this study. This work was supported by a Wellcome Trust Programme Grant to D.B.Y. and by The Wellcome Trust functional genomics Development Initiative (Grant No. 062511) to P.D.B. We also acknowledge BμG@S (the Bacterial Microarray Group at St George's) and The Wellcome Trust for funding their work (**http://www.sghms.ac.uk/depts/medmicro/bugs/**).

### References

1. Arnold-Schild D, Hanau D, Spehner D, *et al.* 1999. Cutting edge: receptor-mediated endocytosis of heat shock proteins by professional antigen-presenting cells. *J Immunol* **162**: 3757.
2. Asea A, Kraeft SK, Kurt-Jones EA, *et al.* 2000. HSP70 stimulates cytokine production through a CD14-dependent pathway, demonstrating its dual role as a chaperone and cytokine. *Nature Med* **6**: 435.
3. Basu S, Binder RJ, Ramalingam T, Srivastava PK. 2001. CD91 is a common receptor for heat shock proteins gp96, hsp90, hsp70, and calreticulin. *Immunity* **14**: 303.

Copyright © 2002 John Wiley & Sons, Ltd. 

4. Binder RJ, Han DK, Srivastava PK. 2000. CD91: a receptor for heat shock protein gp96. *Nature Immunol* **1**: 151.
5. Bucca G, Brassington AM, Schonfeld HJ, Smith CP. 2000. The HspR regulon of *Streptomyces coelicolor*: a role for the DnaK chaperone as a transcriptional co-repressor. *Mol Microbiol* **38**: 1093.
6. Bucca G, Ferina G, Puglia AM, Smith CP. 1995. The dnaK operon of *Streptomyces coelicolor* encodes a novel heat-shock protein which binds to the promoter region of the operon. *Mol Microbiol* **17**: 663.
7. Buchmeier NA, Heffron F. 1990. Induction of *Salmonella* stress proteins upon infection of macrophages. *Science* **248**: 730.
8. Castellino F, Boucher PE, Eichelberg K, *et al.* 2000. Receptor-mediated uptake of antigen/heat shock protein complexes results in major histocompatibility complex class I antigen presentation via two distinct processing pathways. *J Exp Med* **191**: 1957.
9. Chen W, Syldath U, Bellmann K, Burkart V, Kolb H. 1999. Human 60 kDa heat-shock protein: a danger signal to the innate immune system. *J Immunol* **162**: 3212.
10. Cho BK, Palliser D, Guillen E, *et al.* 2000. A proposed mechanism for the induction of cytotoxic T lymphocyte production by heat shock fusion proteins. *Immunity* **12**: 263.
11. Cole ST, Brosch R, Parkhill J, *et al.* 1998. Deciphering the biology of *Mycobacterium tuberculosis* from the complete genome sequence. *Nature* **393**: 537.
12. Fernandes ND, Wu QL, Kong D, Puyang X, Garg S, Husson RN. 1999. A mycobacterial extracytoplasmic sigma factor involved in survival following heat shock and oxidative stress. *J Bacteriol* **181**: 4266.
13. Garbe TR, Hibler NS, Deretic V. 1999. Response to reactive nitrogen intermediates in *Mycobacterium tuberculosis*: induction of the 16 kDa $\alpha$-crystallin homolog by exposure to nitric oxide donors. *Infect Immun* **67**: 460.
14. Hartl FU. 1996. Molecular chaperones in cellular protein folding. *Nature* **381**: 571.
15. Hecker M, Schumann W, Volker U. 1996. Heat-shock and general stress response in *Bacillus subtilis*. *Mol Microbiol* **19**: 417.
16. Helmann JD. 1999. Anti-sigma factors. *Curr Opin Microbiol* **2**: 135.
17. Huang Q, Richmond JFL, Suzue K, Eisen HN, Young RA. 2000. *In vivo* cytotoxic T lymphocyte elicitation by mycobacterial heat shock protein 70 fusion proteins maps to a discrete domain and is $CD4^{+}$T cell independent. *J Exp Med* **191**: 403.
18. Kohler S, Ekaza E, Paquet JY, *et al.* 2002. Induction of dnaK through its native heat shock promoter is necessary for intramacrophagic replication of *Brucella suis*. *Infect Immun* **70**: 1631.
19. Kol A, Lichtman AH, Finberg RW, Libby P, Kurt-Jones EA. 2000. Cutting edge: heat shock protein (HSP) 60 activates the innate immune response: CD14 is an essential receptor for HSP60 activation of mononuclear cells. *J Immunol* **164**: 13.
20. Lee BY, Horwitz MA. 1995. Identification of macrophage and stress-induced proteins of *Mycobacterium tuberculosis*. *J Clin Invest* **96**: 245.
21. Lehner T, Bergmeier LA, Wang Y, *et al.* 2000. Heat shock proteins generate beta-chemokines which function as innate adjuvants enhancing adaptive immunity. *Eur J Immunol* **30**: 594.
22. Monahan I, Betts J, Banerjee D, Butcher P. 2001. Differential expression of mycobacterial proteins following phagocytosis by macrophages. *Microbiology* **147**: 459.
23. Peetermans WE, Raats CJ, van Furth R, Langermans JA. 1995. Mycobacterial 65 kDa heat shock protein induces tumor necrosis factor alpha and interleukin 6, reactive nitrogen intermediates, and toxoplasmastatic activity in murine peritoneal macrophages. *Infect Immun* **63**: 3454.
24. Qoronfleh MW, Bortner CA, Schwartzberg P, Wilkinson BJ. 1998. Enhanced levels of *Staphylococcus aureus* stress protein GroEL and DnaK homologs early in infection of human epithelial cells. *Infect Immun* **66**: 3024.
25. Raman S, Song T, Puyang X, Bardarov S, Jacobs WR Jr, Husson RN. 2001. The alternative sigma factor SigH regulates major components of oxidative and heat stress responses in *Mycobacterium tuberculosis*. *J Bacteriol* **183**: 6119.
26. Sherman DR, Voskuil M, Schnappinger D, Liao R, Harrell MI, Schoolnik GK. 2001. Regulation of the *Mycobacterium tuberculosis* hypoxic response gene encoding $\alpha$-crystallin. *Proc Natl Acad Sci USA* **98**: 7534.
27. Srivastava PK, Menoret A, Basu S, Binder RJ, McQuade KL. 1998. Heat shock proteins come of age: primitive functions acquire new roles in an adaptive world. *Immunity* **8**: 657.
28. Stewart GR, Snewin VA, Walzl G, *et al.* 2001. Overexpression of heat-shock proteins reduces survival of *Mycobacterium tuberculosis* in the chronic phase of infection. *Nature Med* **7**: 732.
29. Suto R, Srivastava PK. 1995. A mechanism for the specific immunogenicity of heat shock protein-chaperoned peptides. *Science* **269**: 1585.
30. Suzue K, Zhou X, Eisen HN, Young RA. 1997. Heat shock fusion proteins as vehicles for antigen delivery into the major histocompatibility complex class I presentation pathway. *Proc Natl Acad Sci USA* **94**: 13146.
31. Tusher VG, Tibshirani R, Chu G. 2001. Significance analysis of microarrays applied to the ionizing radiation response. *Proc Natl Acad Sci USA* **98**: 5116.
32. Vabulas RM, Ahmad-Nejad P, da Costa C, *et al.* 2001. Endocytosed HSP60s use toll-like receptor 2 (TLR2) and TLR4 to activate the toll/interleukin-1 receptor signaling pathway in innate immune cells. *J Biol Chem* **276**: 31332.
33. Vabulas RM, Ahmad-Nejad P, Ghose S, Kirschning CJ, Issels RD, Wagner H. 2002. HSP70 as endogenous stimulus of the Toll/interleukin-1 receptor signal pathway. *J Biol Chem* **277**: 15107.

Copyright © 2002 John Wiley & Sons, Ltd. 
